# Sarcoidosis presenting as bilateral lacrimal gland swelling: a pediatric case report

**DOI:** 10.1186/s12969-021-00606-1

**Published:** 2021-08-06

**Authors:** Margaret S. Powell, Ashley W. Cross, Jared Tallo, Edward W. Cheeseman, Mileka R. Gilbert

**Affiliations:** 1 Department of Ophthalmology & Visual Sciences, University of Wisconsin, Madison, WI USA; 2 Department of Pathology & Laboratory Medicine, Medical University of South Carolina, Charleston, SC USA; 3 College of Medicine, Medical University of South Carolina, Charleston, SC USA; 4 Storm Eye Institute, Department of Ophthalmology, Medical University of South Carolina, Charleston, SC USA; 5 Division of Pediatric Rheumatology, Department of Pediatrics, Medical University of South Carolina, Charleston, SC USA

**Keywords:** Pediatric sarcoidosis, Lacrimal gland, Granulomatous inflammation

## Abstract

**Background:**

To describe a case of pediatric sarcoidosis which initially presented as papillary conjunctivitis before manifesting as bilateral lacrimal gland swelling without other known systemic involvement.

**Case presentation:**

A 10-year-old female presented to the pediatric ophthalmology clinic with complaints of bilateral eyelid swelling, tearing and itching for approximately 1 month. Her history and exam were most consistent with allergic conjunctivitis, for which she was started on a standard topical regimen. Despite initial improvement, she re-presented with significantly worsened eyelid swelling and minimal allergic symptoms. Enlargement of the lacrimal glands were palpable at this time. Lacrimal gland biopsy was obtained which demonstrated noncaseating granulomas. Systemic workup did not reveal evidence of disease involvement elsewhere.

**Conclusions:**

Sarcoidosis in the pediatric population may present in a myriad of ways and is well-known to mimic other disease entities. We present a case of pediatric sarcoidosis which presented initially as papillary conjunctivitis before manifesting as bilateral lacrimal gland swelling without systemic involvement.

## Background

Sarcoidosis is a multisystem disease characterized by granulomatous inflammation. Triggers of sarcoidosis flare with the T-cell-mediated inflammatory response are not known. Genetic associations and infectious or environmental triggers have been posited, however definitive links have not been determined. While pulmonary and skin involvement are most common in adult presentations of sarcoidosis, ocular involvement occurs in 25–60% of patients [1]. Hallmarks of ocular involvement include anterior uveitis, iris nodules, conjunctival nodules; lacrimal glands and orbital adnexa are also frequently affected.

The disease is typically diagnosed in adulthood; however, it may present at any age. Early-onset childhood sarcoidosis (onset at less than 5 years) is distinguished from later-onset or adult-type childhood sarcoidosis, which often demonstrates pulmonary and other systemic involvement. Blau syndrome, which may represent the same disease as early-onset childhood sarcoidosis, is an autosomal-dominant monogenic granulomatous syndrome associated with mutation in the nucleotide binding oligomerization domain containing 2 (*NOD2*) gene that causes the classic triad of uveitis, rash, and polyarthritis with onset in the first few years of life [2]. Pediatric sarcoidosis, though rare, often demonstrates eye involvement in addition to systemic findings. Ocular involvement may occur up to two to three times more frequently in children than in adult patients with sarcoidosis [3]. Blau syndrome accounts for a considerable percentage of ocular involvement in pediatric sarcoidosis [2].

Tissue diagnosis remains the gold standard to confirm the disease. Demonstration of noncaseating granulomas in the appropriate clinical setting and in the absence of other causative factors provides the best support for what is fundamentally a diagnosis of exclusion. Radiographic and laboratory testing such as angiotensin-converting enzyme (ACE) may provide additional support, but lack the sensitivity and specificity for definitive diagnosis [1, 4].

## Case presentation

A ten-year-old African-American female presented to the pediatric ophthalmology clinic in referral from her primary care provider for evaluation of bilateral eyelid swelling, tearing and itching for approximately one month. Her past medical history included premature birth, jejunal atresia with resection and jejuno-ileal primary anastomosis, short gut syndrome, asthma, and mild eczema. The patient’s birth history was complicated by Human Immunodeficiency Virus (HIV) positive testing of her mother, but with normal prenatal ultrasounds and negative congenital HIV testing, and was otherwise uncomplicated. Her family history included allergic rhinitis and asthma in her mother, and there was no known family history of sarcoidosis or granulomatous disease. The patient’s asthma was first suspected when she presented at seven months old with wheezing and cough that responded well to albuterol concerning for reactive airway disease. She is now followed by a pediatric pulmonologist for moderate persistent asthma with pulmonary function tests that revealed a moderate obstruction pattern with normal lung volumes and diffusing capacity of lung for carbon monoxide. She is treated with an inhaled corticosteroid for asthma control and albuterol as needed for symptom exacerbation.

Her presenting ophthalmologic exam was notable for bilateral papillary conjunctivitis and mild superficial keratopathy, as well as mild boggy lid edema. She was started on ketotifen fumarate and artificial tears for presumed allergic conjunctivitis. Five months later at her follow-up exam, she had persistent symptoms and exam with giant papillary reaction of the palpebral conjunctiva of both upper eyelids. Upon referral to an allergist, she was found to have normal serum IgE level, and skin testing did not identify a likely allergen exposure. She had been diagnosed with eczema clinically in the past by her primary care provider and was confirmed by allergist. Inflammatory markers were elevated including sedimentation rate 38 mm/hr. (reference range 0–20 mm/hr) and C-reactive protein 4.18 mg/dL (reference range 0–1 mg/dL); however, serum ACE and lysozyme were normal, and chest radiograph did not reveal hilar lymphadenopathy. At this point, the worsening palpebral reaction and overall picture including history of atopic disease were felt to best support a diagnosis of vernal keratoconjunctivitis. She was started on a topical regimen including corticosteroid, olopatadine and tacrolimus ointment with mild improvement in lid swelling.

**Fig. 1:**
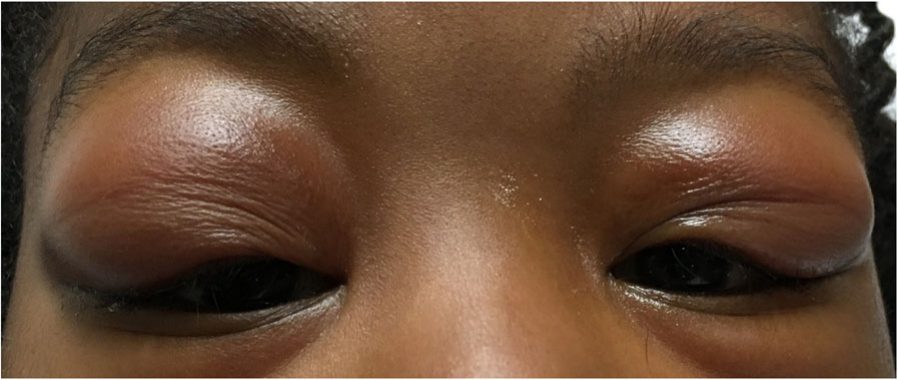
Photograph of our patient showing bilateral temporal upper eyelid swelling

She was next seen approximately 6 weeks later when she presented to the pediatric emergency department with significant worsening of bilateral upper eyelid swelling (Fig. 1). Her exam continued to demonstrate giant papillary reaction temporally in the bilateral superior palpebral conjunctiva. Of note, the upper lid swelling was much more pronounced than at previous visits and the lacrimal glands were palpable bilaterally. She had continued to use topical steroid, tacrolimus, and artificial tears, but had discontinued olopatadine as she had minimal to no itching. Decision was made to pursue tissue diagnosis by obtaining a lacrimal gland biopsy. She was also referred to pediatric rheumatology for evaluation as she complained of joint pains at her emergency department visit. The onset of the joint pain was around the same time as the patient’s swollen eyelids, however there was no joint effusion, limitation, or tenderness on exam. Subsequent radiographs of bilateral ankles and elbows also showed no joint effusion or signs of chronic arthritis. Exam did show bilateral flexible pes planus which likely contributed to patient’s lower extremity pain with high impact weight-bearing activity. She had dry skin on exam without ichthyosiform cutaneous manifestations that can be seen in children with sarcoidosis [2]. There were no discrete lesions or papules, and thus skin biopsy was not pursued. There was also no unexplained fever, weakness, alopecia, sicca symptoms, or oral ulcers by history or exam. Extensive lab evaluation showed normal/negative complete blood count, liver, kidney, and thyroid function, creatine kinase, complement studies (C3, C4, CH50), anti-nuclear antibody, anti-neutrophil cytoplasmic antibody, and urinalysis. Sedimentation rate was persistently elevated at 25 mm/hr. Genetic testing for the *NOD2* gene mutation associated with Blau syndrome was not performed due to lack of uveitis, rash, and arthritis seen in this disease.

**Fig. 2:**
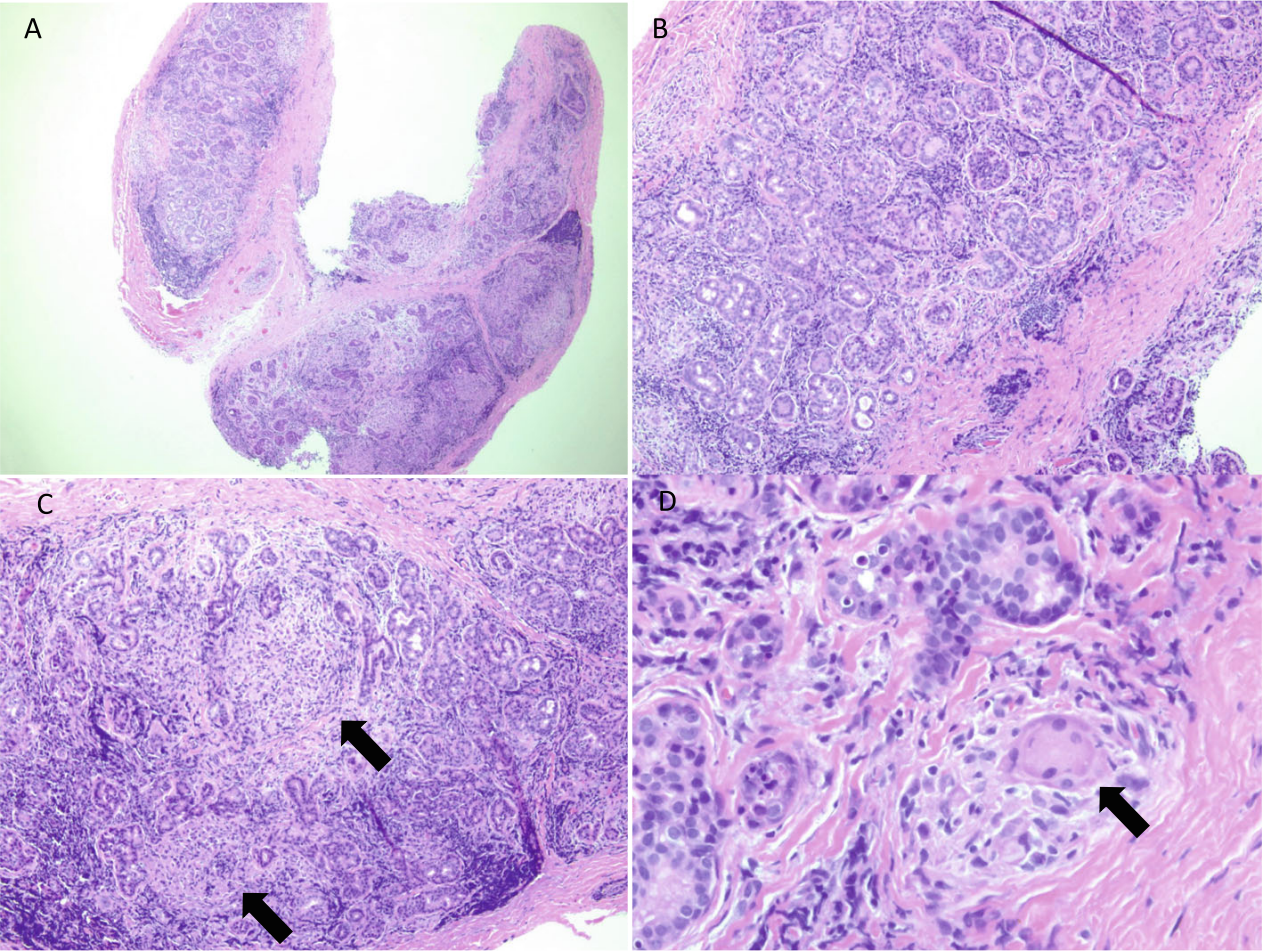
A) 2X and B) 4X hematoxylin and eosin-stained sections of right lacrimal gland biopsy from our patient showing a lymphocytic and granulomatous inflammatory infiltrate. C) 4X Arrows highlight noncaseating granulomas. D) 40X Arrows highlight multinucleated giant cell within granulomatous inflammation (hematoxylin-eosin stain)

**Fig. 3:**
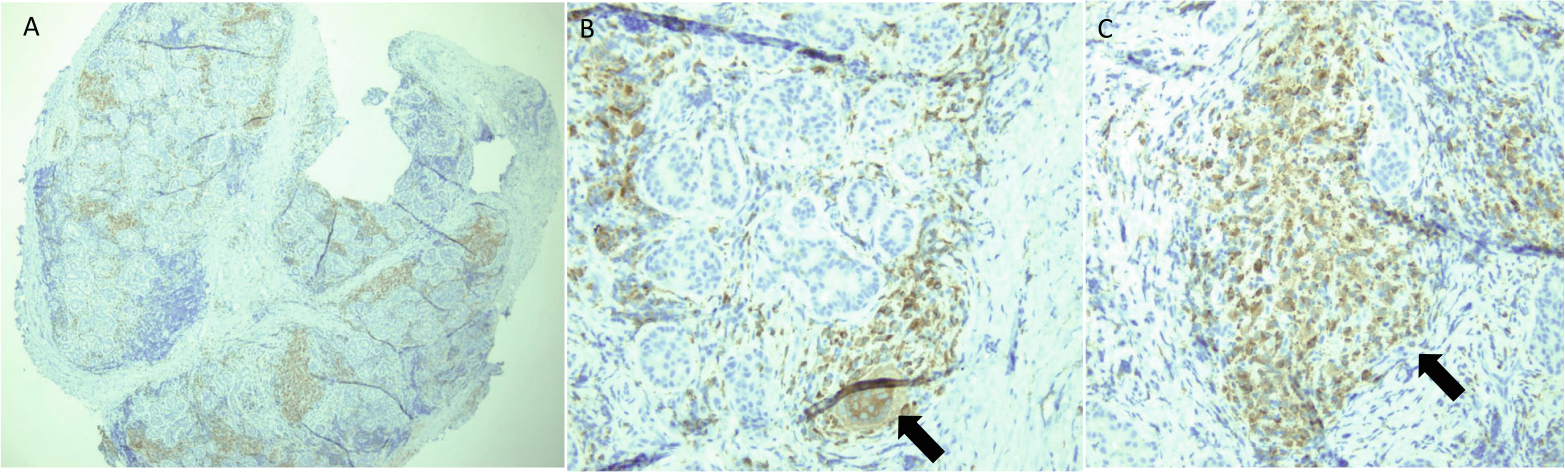
A) 2X and B) 10X sections of right lacrimal gland immunohistochemically stained with CD68. Arrow highlights multinucleated giant cell. C) 10X section highlighting histiocyte aggregates

Eight months after initial presentation, biopsy of the right lacrimal gland was performed in an anterior transseptal fashion through the temporal upper lid crease. Histopathologic evaluation of the lacrimal gland specimen demonstrated chronic-appearing inflammatory changes with poorly-formed noncaseating granulomas and widespread destruction of normal acini and gland structures. Staining for CD68 marker for epithelioid cells showed multiple foci of macrophage infiltration at sites of granulomatous inflammation consistent with sarcoidosis (Figs. 2 and 3). Special stains including Kinyoun stain for acid-fast bacteria and Giemsa stain for fungal organisms were negative.

She was started on treatment with oral prednisone ~ 2 mg/kg/day at the same time of the lacrimal gland biopsy 8 months after initial presentation with gradual improvement in upper eyelid swelling. Subcutaneous methotrexate 15 mg/m2 weekly was added to therapy 6 weeks later as a steroid-sparing agent, and she was able to taper off steroid over the next 2 months. Serial slit lamp exams have demonstrated no evidence of intraocular inflammation, iris nodules, or vascular changes. At the patient’s last visit, 28 months out from initial presentation, she had no other systemic manifestations of sarcoidosis and was doing well on methotrexate.

## Discussion

Anterior uveitis is by far the most common ocular complication of sarcoidosis in both early- and late-onset childhood sarcoidosis, with estimated prevalence of 21–58% in childhood-onset sarcoidosis [2, 5]; however, this was not seen in our patient. Conjunctival nodules have been commonly reported in pediatric as in adult disease. Our case represents the first report to our knowledge of giant papillary conjunctivitis at presentation with subsequent lacrimal gland inflammation consistent with sarcoidosis. Our patient is unique in that she lacks the classic triad of uveitis, rash, and arthritis demonstrated in early childhood-onset sarcoidosis and Blau syndrome. She also lacks pulmonary involvement seen in adult-type childhood-onset sarcoidosis. Of note, review of pathology report from small bowel resection and jejuno-ileal primary anastomosis procedure as an infant showed benign calcifications and fibromuscular connective tissue without granulomatous inflammation.

Sarcoidosis presenting with ocular involvement alone makes the diagnosis a difficult one without tissue biopsy. Intraocular disease involvement is characterized by the International Workshop on Ocular Sarcoidosis (IWOS) criteria [6]; however, there is no similar set of accepted criteria that includes orbital or adnexal involvement. Additionally, IWOS criteria are not validated in children. Low sensitivity and specificity limit the diagnostic value of radiographic and laboratory testing alone. Despite vague complaints of systemic symptoms including subjective fevers and intermittent joint pains, chest and joint radiographs and serum ACE and lysozyme were normal in our patient. Sarcoidosis is characterized by the presence of noncaseating granulomatous inflammation without the presence of foreign bodies or infectious organisms. Pathology shows inflammatory infiltrates that include epithelioid histiocytes (transformed or activated macrophages) as well as lymphocytes; multinucleated giant cells are frequently seen (Figs. 2 and 3). Epithelioid histiocytes may be demonstrated by special stains such as CD68 as seen in our patient.

The lacrimal glands are the most commonly involved orbital structures in sarcoidosis, with reported prevalence of 42–63% in studies of orbital and ocular adnexal sarcoidosis [7–9], and 7–16% in systemic disease [9]. Lacrimal gland involvement has been reported in pediatric sarcoidosis [2, 10–12], but our literature search found no published estimates of prevalence based on histopathologic diagnosis. Granulomatous disease of the lacrimal glands without systemic involvement has also been described in adults, but there is no literature to estimate prevalence of this occurrence in children.

Review of the literature shows that lacrimal gland sarcoidosis associated with systemic disease is variable. Lacrimal gland biopsies performed at Wills Eye Hospital demonstrated histopathology consistent with sarcoidosis in 20% of patients without known systemic disease [13]. Of these patients 33% had elevated ACE levels, although over half (53%) did not have documented serum ACE results. Another study by *Demirci* et al found that only 37% of biopsy-proven orbital and adnexal sarcoidosis patients in their series had known systemic disease; over the course of follow-up, only half of these patients subsequently developed evidence of systemic disease [9]. *Mavrikakis* et al described 20 patients, in 18 (90%) of whom orbital inflammation was the presenting symptom of sarcoidosis, with 2/20 (10%) demonstrating anterior uveitis prior to development of orbital symptoms. Over half (11/20, 55%) had lacrimal gland infiltration. Although 19/20 patients had histopathology consistent with sarcoidosis on orbital biopsy, only 50% subsequently demonstrated systemic disease, and of these 60% were asymptomatic [8]. Another case series of lacrimal gland sarcoidosis published by *Yanardag* et al found that 9 of 516 (1.74%) sarcoidosis patients had biopsy-proven lacrimal gland involvement. Five of these patients (55%) had no radiographic evidence of pulmonary disease, and 2 (22%) had no other systemic findings of sarcoidosis. Serum ACE was not measured [10].

While all the studies above described adult or mixed patient populations, *Belanger* et al reported a case series of orbital inflammation in pediatric patients [11]. Two-thirds of the 12 patients in this study presented with lacrimal gland enlargement with three bilateral cases. Two patients were diagnosed with sarcoidosis and presented with bilateral lacrimal gland enlargement; one demonstrated typical systemic symptoms, while the other patient was reportedly asymptomatic though found to have renal involvement. Diagnosis was made via biopsy of lung and kidney respectively (whether they also had lacrimal gland biopsy was not reported). The remaining bilateral case was treated for presumed *Chlamydia* infection based on history and presentation. This literature review of isolated lacrimal gland sarcoidosis at presentation highlights the importance of close monitoring over time for the evolution of systemic disease.

The reason why the lacrimal gland is so frequently involved in sarcoidosis is unclear. Like the parotid gland which is also commonly involved in sarcoidosis, the lacrimal gland is also an exocrine gland and has similar structure with acinar cells and mucus tubules. They both have important functions in lubrication and protection from the external environment. However, genetic and immunological characteristics of these glands that increase susceptibility to sarcoid disease are unclear.

In the absence of systemic findings, some authors debate whether a diagnosis of sarcoidosis can be made. *Mombaerts* et al published a case series of seven patients with orbital granulomatous inflammation, none of whom had systemic evidence of sarcoidosis at presentation or at follow-up (study average 9.5 years). All cases were unilateral, and most presented with pain. Patient age ranged from three to 65 years. The authors classified these patients as representative of a “granulomatous variant of orbital pseudotumor.” In this study, the authors argued against labeling isolated orbital granulomatous disease as a limited or forme fruste sarcoidosis on the basis of both non-granulomatous and granulomatous components of inflammation in the studied specimens, as well as unilateral presentation, extralacrimal inflammation (not involving optic nerve or extraocular muscles), and normal serum ACE levels [14]. Orbital pseudotumor, or nonspecific orbital inflammation, is well-known to be bilateral much more frequently in children than in adults. However, it is characteristically described as a polymorphous fibroinflammatory infiltrate, occasionally with sclerotic components [15]. Response to corticosteroid therapy is typically prompt. In our patient, the bilateral, subacute lacrimal gland enlargement without significant periorbital pain is less consistent with orbital pseudotumor, as is her gradual improvement on corticosteroids followed by methotrexate.

## Conclusions

While bilateral lacrimal gland enlargement has been described as a presenting sign of sarcoidosis in adults, this presentation in pediatric patients is less well-represented in the literature. This case is unusual for a presentation of significant bilateral lacrimal gland swelling with histopathologic evidence of noncaseating granulomas in the absence of definitive systemic signs of sarcoidosis. Additionally, the presentation of giant papillary conjunctivitis with subsequent lacrimal gland inflammation consistent with sarcoidosis has not been reported in the literature. Although it is certainly possible that our patient suffered from allergic conjunctivitis simultaneously with sarcoid-associated lacrimal gland inflammation, it is noteworthy that with worsening lacrimal gland inflammation, she had persistent giant papillary reaction in the temporal palpebral conjunctiva, without significant allergic symptoms. This case agrees with the existing literature in that sarcoidosis can have diverse presentations, and tissue diagnosis may be essential to diagnose even if laboratory and radiographic evidence are lacking. Systemic disease may develop late so long-term monitoring and interdisciplinary management are crucial to optimize outcomes.

## Data Availability

Not applicable.

## Abbreviations

NOD2: Nucleotide binding oligomerization domain containing 2
ACE: Angiotensin-converting enzyme
HIV: Human Immunodeficiency Virus
IWOS: International Workshop on Ocular Sarcoidosis
